# Emerging immune networks and targeted strategies in T2 asthma

**DOI:** 10.3389/fimmu.2026.1868787

**Published:** 2026-07-17

**Authors:** Tianye Xi, Tingting Zu, Xinru Pang, Fuling Wu

**Affiliations:** Shandong Medical and Pharmaceutical University Hospital, Binzhou, China

**Keywords:** biologics, ferroptosis, group 2 innate lymphoid cells, interleukin-33, precision medicine, T2 asthma

## Abstract

Asthma is a highly heterogeneous chronic inflammatory disease of the airways, among which Th2-high asthma represents the most prevalent endotype. The pathogenesis of Type 2 (T2) asthma (driven by type 2 inflammation) involves a complex immune network orchestrated by the coordinated actions of multiple effector cell populations, including Th2 cells, group 2 innate lymphoid cells (ILC2s), and type 2 cytotoxic T (Tc2) cells. Epithelial-derived alarmins, particularly interleukin-33 (IL-33) and thymic stromal lymphopoietin (TSLP), function as key upstream initiators that bridge innate and adaptive immunity. In addition, multilayered regulatory mechanisms—including genetic susceptibility, metabolic reprogramming, and ubiquitination—collectively govern the initiation and progression of Th2-driven inflammation. With the deepening understanding of these mechanisms, therapeutic strategies have progressively shifted from targeting downstream effector molecules to upstream alarmins, thereby providing new directions for precision medicine. This review systematically summarizes recent advances in the immunopathogenesis and targeted therapies of T2 asthma, offering a conceptual framework for precision-based clinical interventions.

## 1 Introduction

Asthma is one of the most common chronic respiratory diseases worldwide, characterized by a high prevalence and a substantial global health burden. According to the Global Burden of Disease study, approximately 260 million individuals are currently affected by asthma globally, and the overall prevalence has continued to rise since the beginning of the 21st century (1). Although the age-standardized prevalence of asthma has gradually stabilized in some high-income countries, the incidence continues to show a marked upward trend in many low- and middle-income countries (2). In China, with the acceleration of urbanization and changes in lifestyle, the prevalence of asthma has also shown a rising trend, making it an increasingly important public health concern (3). In addition, asthma not only severely impairs patients’ quality of daily life but also imposes a substantial social and economic burden. For example, in the United States, annual healthcare expenditures related to asthma have exceeded $80 billion, and the disability-adjusted life years (DALYs) attributable to asthma account for more than 1% of the total disease burden (2), underscoring the urgency of improving disease prevention and therapeutic strategies.

Historically, asthma was regarded as a single disease entity, and therapeutic strategies were relatively uniform, primarily relying on inhaled corticosteroids and bronchodilators for symptom control. However, with advances in molecular immunology and high-throughput technologies, asthma is now increasingly recognized as a heterogeneous syndrome driven by multiple immune pathways, leading to a paradigm shift in its diagnosis and management. Based on the molecular characteristics of airway inflammation, asthma is currently broadly classified into Th2-high and Th2-low endotypes. Among these, Th2-high asthma accounts for approximately 50%–70% of cases and is particularly prevalent in pediatric populations and in early-onset disease (1). The hallmark of this endotype is the aberrant activation of type 2 immune responses, typically characterized by eosinophilic infiltration, elevated levels of immunoglobulin E (IgE), increased fractional exhaled nitric oxide (FeNO), as well as airway hyperresponsiveness and excessive mucus production (4).

Based on advances in the understanding of the immunological mechanisms underlying T2 asthma, this review systematically summarizes its immunopathogenesis and recent progress in targeted therapies, focusing on key immune cell populations, critical regulatory pathways, potential therapeutic targets, and future research directions, thereby providing a theoretical framework for precision medicine in asthma.

## 2 Core immune cells and infiammatory regulatory networks in asthma

Historically, Th2 cells have been considered the primary effector cells driving the inflammatory response in T2 asthma. With the deepening understanding of asthma immunology, it has become increasingly evident that, in addition to Th2 cells, group 2 innate lymphoid cells (ILC2s) and type 2 cytotoxic T cells (Tc2 cells) also contribute to the initiation and progression of the disease.

These three cell populations differ in their origins, activation pathways, and functional characteristics; however, they exhibit pronounced synergistic effects during the development of airway inflammation. They participate in immune responses at distinct stages and, through the secretion of a range of type 2 cytokines, collectively drive the initiation and maintenance of Th2-mediated inflammation (**Table 1**).

**Table 1:** Pharmacological and functional heterogeneity of core type 2 immune effector cells in Th2-high asthma.

Feature	Th2 cells	ILC2	Tc2 cells
Lineage	Adaptive immune (CD4^+^ T cells)	Innate lymphoid cells	Adaptive immune (CD8^+^ T cells)
Differentiation/Origin	Naïve CD4^+^ T cells differentiate upon antigen stimulation	Derived from common lymphoid progenitors; develop under IL-7 and other cytokines	Naïve CD8^+^ T cells differentiate upon antigen stimulation
Activation mechanisms	TCR recognition of antigen–MHC II complexes + cytokines (e.g., IL-4)	Activated by epithelial-derived alarmins (IL-33, IL-25, TSLP)	TCR recognition of antigen–MHC I complexes + cytokines
Key transcription factors	GATA3	GATA3, RORα	GATA3
Major effector cytokines	IL-4, IL-5, IL-13	IL-5, IL-13, IL-9, amphiregulin	IL-5, IL-13, low IL-4, IFN-γ (context-dependent)
Functional roles	Drive eosinophilic inflammation, IgE class switching, airway remodeling	Rapid initiation of type 2 immunity, tissue repair, amplification of inflammation	Cytotoxicity combined with type 2 inflammation; potential role in chronic inflammation
Role in asthma	Central effector cells sustaining chronic inflammation and airway hyperresponsiveness	Early initiators; associated with severe and steroid-resistant asthma	Emerging contributors; increased in subsets of patients (e.g., virus-associated asthma)

Th2, T helper 2; ILC2, group 2 innate lymphoid cells; Tc2, type 2 cytotoxic T cells; GATA3, GATA binding protein 3; RORα, RAR-related orphan receptor alpha; IL, interleukin. a The activation of ILC2s does not rely on classical antigen-presenting mechanisms but is rapidly triggered by epithelial-derived alarmins. b Tc2 cells demonstrate remarkable plasticity and may exhibit a mixed Tc1/Tc2 phenotype during viral-induced asthma exacerbations.

### 2.1 Heterogeneity and functional characteristics of pathogenic Th2 cells

As a key cellular component of adaptive immunity, Th2 cells are among the earliest effector populations identified in asthma research. Classical studies have shown that naïve CD4^+^ T cells differentiate into Th2 cells under the influence of interleukin-4 (IL-4) via the STAT6/GATA3 signaling axis, leading to the production of canonical Th2 cytokines, including IL-4, IL-5, and IL-13, thereby promoting the development of allergic inflammation (5).With the widespread application of single-cell sequencing technologies, Th2 cells are now recognized as a heterogeneous population composed of multiple functionally distinct subsets rather than a uniform cell type. Using a conditional knockout mouse model targeting histone deacetylase 1 (HDAC1), combined with single-cell transcriptomic analysis, Khan et al. systematically characterized Th2 cells in lung tissue and identified at least two major pathogenic subsets, namely pathogenic effector Th2 (peTh2) cells and Th2 tissue-resident memory (Th2 Trm) cells. In terms of phenotypic features, peTh2 cells typically exhibit high expression of IL-4, IL-5, IL-13, and members of the tumor necrosis factor receptor superfamily(TNFRSF) family (such as OX40, 4-1BB, and GITR), reflecting potent pro-inflammatory capacity, whereas Th2 Trm cells display characteristic tissue-resident properties, including high CD69 expression and low levels of sphingosine-1-phosphate receptor 1 (S1pr1) and CC chemokine receptor 7 (CCR7). Further transcriptomic analyses suggest that although these two subsets share partial overlap in the expression of pathogenic genes, they exhibit marked differences in metabolic states and effector molecule profiles (6). Subsequent studies have further substantiated the existence of such Th2 subset heterogeneity. Zou et al., through single-cell transcriptomic analysis of CD4^+^ T cells from patients with asthma and chronic rhinosinusitis, demonstrated that hypoxia-inducible factor 2α (HIF-2α) is selectively upregulated in pathogenic Th2 cells and cooperates with GATA3 to regulate the expression of multiple downstream pathogenic genes. Trajectory analysis further suggested that HIF-2α may play a critical regulatory role in the transition from stem-like Th2 cells to pathogenic Th2 subsets (7). In addition, emerging evidence indicates that the complement system is also involved in the regulation of type 2 immune responses. Thomas et al. highlighted that the complement system, beyond being a key component of innate immunity, can modulate the initiation and amplification of Th2 responses by influencing the function of dendritic cells, ILC2s, and T cells. In particular, C3, C5, and their activation fragments play critical roles in the context of allergic inflammation (8).

Accurate identification and isolation of pathogenic Th2 (pTh2) subsets are essential for elucidating their functional properties. Khan et al. established a surface marker–based sorting strategy in which ST2^+^CD27^-^KLRG1^-^ cells can be used to effectively enrich pTh2 populations; further discrimination between pathogenic effector Th2 (peTh2) cells and Th2 tissue-resident memory (Th2 Trm) cells can be achieved by combining CD69 and PD-1 expression, with peTh2 defined as CD69^lo PD-1^hi and Th2 Trm as CD69^hi PD-1^hi. This strategy provides an important tool for subsequent functional studies and also lays the groundwork for potential clinical detection and diagnostic applications (6). Pizzarello et al. also focused on the early identification of pathogenic Th2 populations and described a novel effector memory Th2 subset, termed Th2B cells (CD25^+^CD127^+^CD161^-^CD49d^+^CCR4^+^CRTH2^+^), which was significantly increased in urban high-risk infants at 6 months of age and was associated with the subsequent development of allergic diseases (9). Notably, this cell population exhibits high expression of Th2-associated molecules such as GATA3 and CRTH2, and shares similar transcriptional features and pro-inflammatory functions with the peTh2 cells reported by Khan et al. These findings suggest that Th2B cells may represent a developmentally specific phenotype of pathogenic Th2 (pTh2) cells in early human life, and that their identification and isolation strategies hold promise for screening high-risk populations and enabling early intervention in asthma.

Pathogenic Th2 (pTh2) cells play a pivotal role in the initiation and persistence of airway inflammation, with their pathogenic effects largely mediated by the secretion of multiple type 2 cytokines. Among these, interleukin-5 (IL-5) and interleukin-13 (IL-13) are considered the most critical effector molecules. IL-5 promotes the differentiation, recruitment, and activation of eosinophils, whereas IL-13 primarily acts on airway epithelial cells to induce goblet cell hyperplasia and increased mucus production, thereby exacerbating airway inflammation. In addition to canonical Th2 cytokines, pTh2 cells can also produce a range of mediators involved in airway remodeling. For instance, Morimoto et al. reported that a subset of pTh2 cells is capable of secreting amphiregulin (Areg), which induces eosinophils to produce osteopontin, thereby promoting airway fibrosis and structural remodeling and contributing to the chronic progression of asthma (10). In addition, alarmin signaling within the inflammatory microenvironment can markedly enhance the pathogenic potential of pTh2 cells. Endo et al. demonstrated that interleukin-33 (IL-33) can promote the acquisition of a more pathogenic phenotype in memory Th2 cells via activation of the p38 MAPK signaling pathway. Upon re-exposure to allergens, these cells rapidly produce large amounts of effector cytokines, thereby amplifying the inflammatory response (11). Further single-cell transcriptomic analyses have also revealed distinctive molecular features of pathogenic Th2 (pTh2) cells. Using single-cell RNA sequencing, Tibbitt et al. demonstrated that pTh2 cells commonly exhibit high expression of the IL-33 receptor ST2 and the metabolic regulator peroxisome proliferator-activated receptor gamma (PPARγ). Notably, PPARγ plays a critical role in maintaining pTh2 cell function and inflammatory capacity; as a key master of energy and lipid pathways, it modulates the metabolic state of immune cells to sustain their activation, proliferation, and cytokine production. These findings suggest that metabolic reprogramming, whereby immune cells remodel their metabolic profiles to meet functional demands, serves as a fundamental mechanism governing pTh2 cell activity (12).

### 2.2 Pathogenic roles of group 2 innate lymphoid cells

Group 2 innate lymphoid cells (ILC2s) have emerged as a key innate immune population receiving increasing attention in asthma research in recent years (4). In contrast to classical adaptive immune cells, ILC2s do not rely on antigen-specific receptors for antigen recognition but are primarily activated by sensing multiple epithelial-derived “alarmin” signals. These cells are abundantly distributed in mucosal tissues, particularly within the respiratory tract, and therefore play an important role in airway inflammatory responses (13). Upon stimulation by allergens, viral infections, or air pollutants, damaged airway epithelial cells release cytokines such as interleukin-33 (IL-33), interleukin-25 (IL-25), and thymic stromal lymphopoietin (TSLP). These epithelial-derived signals rapidly activate ILC2s and induce the production of large amounts of type 2 cytokines, including IL-5 and IL-13, thereby promoting eosinophil recruitment, increased mucus secretion, and the development of airway hyperresponsiveness (14). Because the activation of ILC2s does not depend on classical antigen presentation mechanisms or require conventional T cell differentiation, these cells are widely regarded as an important source initiating early type 2 inflammatory responses. Upon stimulation of airway epithelial cells and subsequent release of alarmin signals, ILC2s can be rapidly activated and produce a range of type 2 cytokines, thereby participating in the early phase of airway inflammation. Notably, multiple asthma genome-wide association studies have identified interleukin 1 receptor-like 1 (IL1RL1) as one of the most significant and consistently replicated susceptibility loci. IL1RL1 encodes the IL-33 receptor ST2, and this genetic evidence further underscores the critical role of the IL-33/ST2 signaling axis in the pathogenesis of asthma, while also highlighting the central contribution of ILC2s in Th2-mediated inflammation (15).

Upon activation, ILC2s rapidly exert potent pro-inflammatory functions. Activated ILC2s promptly produce large amounts of IL-5 and IL-13, at a much faster rate than Th2 cells, as they do not require clonal expansion or differentiation. IL-5 is a key regulator of eosinophil differentiation, activation, and survival; by binding to the interleukin-5 receptor alpha (IL-5Rα) expressed on eosinophils, it activates the Janus kinase/signal transducer and activator of transcription (JAK/STAT) signaling pathway, thereby promoting their release from the bone marrow and subsequent migration to the airways (16, 17). IL-13 can directly act on airway epithelial cells to induce mucin 5AC (MUC5AC) expression, leading to mucus hypersecretion; it also promotes collagen deposition and fibrosis by stimulating fibroblasts, and can directly act on airway smooth muscle to enhance contractility, thereby mediating airway hyperresponsiveness (18). This rapid-response capability positions ILC2s as a “first responder” population in Th2 inflammation, playing a critical role in the early phase of inflammatory responses and in acute exacerbations.

Notably, ILC2s exhibit distinct features in steroid-resistant asthma. In recent years, the identification of inflammatory ILC2s has represented a major advance in this field. van der Ploeg et al. reported that, under stimulation with IL-33 and STAT5-inducing cytokines (such as IL-2 and IL-7), resting CD45RA^+^ ILC2s can differentiate into CD45RO^+^ inflammatory ILC2s. These cells display enhanced proliferative capacity and increased cytokine production; importantly, they exhibit marked resistance to glucocorticoids. In patients with severe asthma and chronic rhinosinusitis with nasal polyps, the proportion of CD45RO^+^ ILC2s is significantly elevated and positively correlates with disease severity (19). Kim et al. further elucidated the molecular mechanisms underlying ILC2-mediated steroid resistance: in an ovalbumin (OVA)/IL-33–induced steroid-resistant asthma mouse model, ILC2s acquired glucocorticoid resistance whereas Th2 cells remained steroid-sensitive, suggesting fundamental differences in their signaling pathways. Mechanistic analyses revealed that the JAK3/STAT5 pathway plays a critical role in both the activation of ILC2s and the development of steroid resistance. Notably, this study also identified a multipotent ILC2 subset (IL-5^+^IL-13^+^IL-17A^+^), which is enriched in steroid-resistant asthma and exhibits sensitivity to JAK3 inhibitors (20).

### 2.3 Noncanonical roles of type 2 cytotoxic T cells

Beyond Th2 cells and ILC2s, type 2 cytotoxic T (Tc2) cells have increasingly come into focus in recent years. Traditionally, CD8^+^ T cells have been regarded primarily as effector cells mediating cytotoxic immunity and have received relatively limited attention in asthma research. However, van der Ploeg et al., through systematic investigations using clinical samples and animal models, demonstrated that this conventional view is being revised by emerging evidence regarding the origin and function of Tc2 cells (13, 19).

The differentiation of Tc2 cells requires the coordinated action of interleukin-4 (IL-4) and interleukin-33 (IL-33). IL-4 drives Tc2 polarization via the STAT6/GATA3 signaling axis, whereas IL-33 enhances their survival and effector functions through ST2-mediated signaling. Chen et al. further demonstrated that Tc2 cells highly express the prostaglandin D_2_ (PGD_2_) receptor DP2 (CRTH2); activation of the PGD_2_/DP2 signaling axis not only promotes Tc2 cell migration, adhesion, and survival, but also induces the production of Th2-type cytokines such as IL-5 and IL-13, and upregulates the expression of a range of genes associated with tissue remodeling (21). Notably, during acute exacerbations of asthma, IL-5^+^ and IL-9^+^ Tc cells can account for approximately 25% of circulating Tc cells, a proportion nearly comparable to that of Th cells (19), suggesting that the role of Tc2 cells in severe asthma and acute exacerbations may have been substantially underestimated.

Of particular interest, a subset of Tc2 cells exhibits a hybrid Tc1/Tc2 phenotype, characterized by the concurrent production of interferon-γ (IFN-γ) and type 2 cytokines such as IL-5, IL-9, or IL-13 (19). This transition from classical antiviral effector cells (Tc1) to a pro-inflammatory Tc2 phenotype highlights the remarkable plasticity of CD8^+^ T cells within chronic inflammatory microenvironments. Such cells may serve as a functional bridge in virus-induced asthma exacerbations: virus-specific Tc1 cells can be reprogrammed toward a Tc2 phenotype under the influence of local IL-4 and IL-33, thereby retaining antiviral activity while acquiring the capacity to promote Th2-mediated inflammation, offering a novel perspective on the link between viral infections and asthma exacerbation.

The clinical relevance of Tc2 cells is becoming increasingly evident. Wirth et al. demonstrated that in patients with severe asthma receiving mepolizumab (a monoclonal antibody targeting IL-5) therapy, the frequency of circulating Tc2 cells is significantly increased, with an increased proportion of the central memory subset (CD62L^+^CCR7^+^) and a decreased proportion of the effector memory subset. This phenotypic remodeling suggests altered homing potential of Tc2 cells from peripheral tissues toward lymphoid organs, which may be associated with disease relapse following treatment discontinuation (22). In addition, a study investigating the origin of atopic predisposition reported that IgG derived from atopic individuals can induce the differentiation of thymocytes from non-atopic infants into Tc2 cells *in vitro*, whereas IgG from non-atopic individuals promotes the generation of IFN-γ^+^ CD8^+^ T cells (23). These findings suggest that immunological imprinting mediated by maternal or endogenous IgG may influence the developmental potential of Tc2 cells early in life, providing new insights into the establishment of atopic predisposition.

### 2.4 Interaction network of Th2, ILC2, and Tc2

In the preceding sections, the origins, activation pathways, functional characteristics, and roles in asthma of Th2 cells, ILC2s, and Tc2 cells have been systematically reviewed. However, these three cell types do not function in isolation *in vivo*. They are temporally and functionally interconnected, forming a synergistic network through complex intercellular interactions that collectively drive the initiation and persistence of Th2-type asthma. Notably, airway epithelial cells serve not only as target cells of inflammatory damage within this network but also as critical hubs bridging innate and adaptive immunity. Allergens, viral infections, and environmental stimuli can induce epithelial cells to release alarmins such as IL-33, TSLP, and IL-25, thereby coordinating the activation and expansion of ILC2s, Th2 cells, and Tc2 cells, while continuously shaping a local microenvironment that favors the development of type 2 inflammation. Therefore, the bidirectional regulatory network formed between epithelial cells and effector lymphocytes represents an essential basis for driving the onset and persistence of Th2-type asthma.

The bidirectional crosstalk between ILC2s and Th2 cells constitutes the core link of this network. In terms of initiation timing, ILC2s are positioned upstream of the inflammatory response, whereas Th2 cells are primarily responsible for the maintenance and amplification of inflammation. Alarmins such as IL-33 and TSLP released by airway epithelial cells can rapidly activate ILC2s, enabling them to produce large amounts of IL-5 and IL-13 within hours, thereby mediating the early inflammatory response (13). Subsequently, ILC2-derived IL-13 can promote the maturation and migration of dendritic cells, driving the differentiation of naive CD4^+^ T cells into Th2 cells (18). Meanwhile, activated Th2 cells act on airway epithelial cells by secreting IL-4, inducing them to produce more TSLP and IL-33, which in turn further activate and expand ILC2s (24). This bidirectional crosstalk forms a positive feedback amplification loop, which is one of the key mechanisms by which Th2-type inflammation is sustained and exacerbated.

The integration of Tc2 cells into this network is equally worthy of attention. The differentiation and survival of Tc2 cells are highly dependent on IL-33 signaling, which establishes a close link between them and both the epithelial-derived alarmin system and the activation pathway of ILC2s (21). In severe asthma and during acute exacerbations, the proportion of Tc2 cells is significantly elevated, and may even be comparable to that of Th2 cells (21). In addition to being closely associated with the alarmin system, Tc2 cells and Th2 cells also exhibit substantial functional overlap in their effector functions. Both are capable of producing large amounts of IL-5 and IL-13, collectively promoting eosinophil recruitment, survival, and airway remodeling (18, 25). Therefore, Tc2 cells do not participate in the inflammatory response independently, but rather serve as an important component of the Th2-type immune network, working together with Th2 cells and ILC2s to drive disease progression (13, 25).

The synergistic effects of the three cell types are particularly prominent in the formation of eosinophilic inflammation. IL-5 is a core factor for the recruitment and survival of eosinophils, and Th2 cells, ILC2s, and Tc2 cells all possess the ability to produce IL-5 (26). Meanwhile, IL-13 derived from Th2 cells and ILC2s can induce airway epithelial cells to produce eotaxin family chemokines, which recruit eosinophils via the CCR3 receptor (27). In severe asthma, this synergistic network also exhibits differential resistance to glucocorticoids: ILC2s can acquire steroid resistance through the JAK3/STAT5 pathway, whereas Th2 cells remain steroid-sensitive (18). This difference suggests that relying solely on glucocorticoids to inhibit Th2 cells may be insufficient to control inflammation driven by ILC2s and Tc2s.

Insights from clinical treatment further validate the importance of this network. In patients with severe asthma receiving mepolizumab (anti-IL-5) therapy, the frequencies of circulating ILC2s, Th2 cells, and Tc2 cells increase synchronously, suggesting that the three cell types coordinately participate in asthma pathology (22). Strategies targeting upstream alarmins, such as the anti-TSLP monoclonal antibody tezepelumab, can simultaneously inhibit the activation of all three cell types at the source, demonstrating broader application prospects (28).

**Figure 1:**
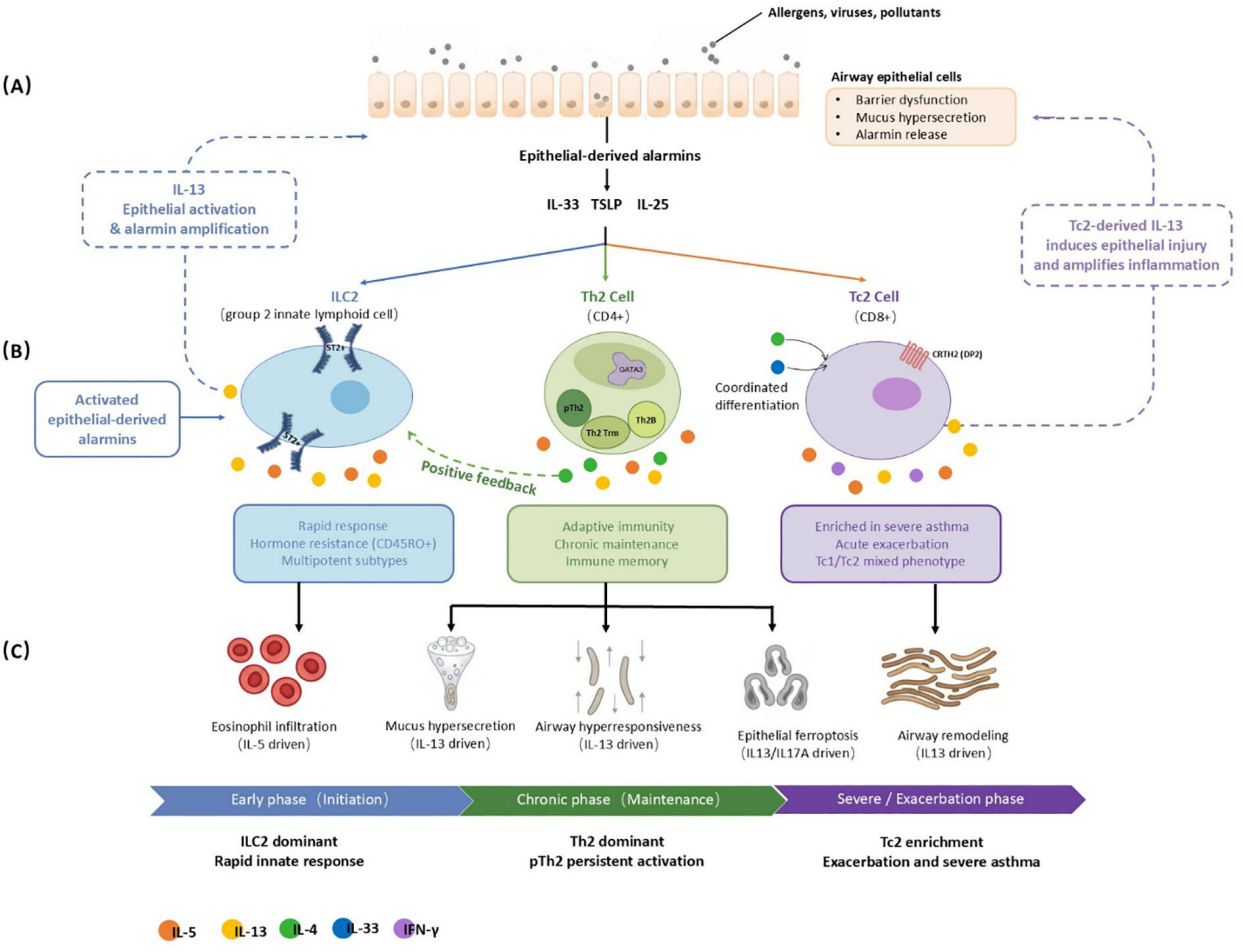
Comprehensive overview of the multicellular immune network and pathogenic signaling pathways in type 2 asthma. **(A)** Upstream Activation and Barrier Injury: Environmental stimuli (allergens, viruses, pollutants) trigger the release of epithelial-derived alarmins (IL-33, TSLP, and IL-25), initiating the downstream inflammatory cascade. **(B)** Multicellular Coordination Axis: Innate and adaptive immune components act synergistically across different chronological phases. Group 2 innate lymphoid cells (ILC2s) represent the “rapid response force” driving acute exacerbations and steroid resistance via the JAK3/STAT5 pathway, leading to the emergence of inflammatory (CD45RO+) and multipotent (IL-5+ IL-13+ IL-17A+) sub-phenotypes. Concurrently, naive CD4+ T cells differentiate into pathogenic Th2 cells (peTh2 and Th2 Trm subsets) to maintain chronic adaptive inflammation. Type 2 cytotoxic T cells (Tc2 cells), driven by coordinated signaling and metabolic reprogramming, further augment eosinophilic infiltration and tissue remodeling during severe stages and acute exacerbations. **(C)** Downstream Pathological Outcomes: The collaborative production of type 2 cytokines (IL-4, IL-5, IL-13) ultimately drives hallmark asthmatic features, including eosinophil recruitment, mucus hypersecretion, airway hyperresponsiveness, tissue remodeling, and epithelial ferroptosis, establishing a vicious feed-forward cycle of airway inflammation. ILC2s primarily drive the early innate phase of T2 inflammation, Th2 cells sustain and amplify chronic adaptive immune responses, whereas Tc2 cells become increasingly enriched during severe disease and acute exacerbations.

In summary, Th2 cells, ILC2s, and Tc2 cells form an interdependent, dynamically regulated multilayered immune network in Th2-type asthma. Among them, ILC2s primarily mediate the initiation of early innate type 2 inflammation, Th2 cells are responsible for the maintenance and amplification of adaptive immune responses, and Tc2 cells further enhance inflammatory responses during disease exacerbation and severe stages. Driven by alarmin signals released by airway epithelial cells, the three cell types interact through cytokine networks and feedback regulatory loops, collectively promoting the onset, persistence, and progression of the disease. An in-depth understanding of the operational principles of this synergistic network will provide important theoretical foundations for the development of combination targeted therapeutic strategies (**Figure 1**).

### 2.5 Modulation of the type 2 inflammatory network by other immune cells

Although Th2 cells, ILC2s, and Tc2 cells are considered the core effector cells driving the initiation and progression of type 2 asthma, a growing body of research indicates that other immune cells also participate in the initiation, amplification, and maintenance of type 2 inflammatory responses (13, 18). By regulating cytokine secretion, promoting immune cell recruitment, and mediating humoral immune responses, these cells together with the Th2–ILC2–Tc2 axis constitute a complex immunoregulatory network.

Innate-like T cells serve as an important bridge connecting innate and adaptive immunity. Among them, invariant natural killer T (iNKT) cells possess characteristics of both T cells and natural killer cells, and can rapidly produce type 2 cytokines such as IL-4 and IL-13 upon activation, thereby promoting Th2 cell polarization and eosinophilic inflammation. Studies have found that iNKT cells show an increasing trend in the airways of some asthma patients, and their activation level is closely correlated with airway hyperresponsiveness and disease severity (29). Mucosal-associated invariant T (MAIT) cells are mainly distributed in the respiratory mucosa and can produce IL-17, IFN-γ, as well as some type 2 cytokines under different inflammatory microenvironments, and are therefore considered to be involved in the regulation of different inflammatory phenotypes of asthma. Recent studies have found that abnormalities in the number and function of MAIT cells are associated with severe asthma and glucocorticoid resistance (30). γδT cells are capable of rapidly sensing epithelial injury and infection signals, and participate in the regulation of airway inflammation by secreting cytokines such as IL-17A and IL-4. Different subsets of γδT cells may exert pro-inflammatory or immunomodulatory effects in asthma, with their specific functions being significantly influenced by the local microenvironment (31).

Dendritic cells (DCs) serve as an important bridge connecting innate and adaptive immunity. Alarmins such as IL-33 and TSLP released by airway epithelial cells can not only directly activate ILC2s, but also promote DC maturation and migration, enhancing their antigen-presenting capacity (18). Activated DCs further induce the differentiation of naive CD4^+^ T cells into Th2 cells, thereby promoting the establishment and maintenance of adaptive type 2 immune responses (18, 32). Therefore, DCs play a critical role in the immunoregulatory network connecting epithelial cells, ILC2s, and Th2 cells.

In addition to cellular immunity, B cells and their mediated IgE responses also constitute an important component of the pathological process in type 2 asthma. Under the action of type 2 cytokines such as IL-4 and IL-13, B cells undergo immunoglobulin class switching and produce specific IgE antibodies (33). After IgE binds to the high-affinity receptor FcϵRI on the surface of mast cells and basophils, it can rapidly induce degranulation upon re-exposure to allergens, releasing histamine, leukotrienes, and various inflammatory mediators, thereby promoting bronchoconstriction, mucus hypersecretion, and airway hyperresponsiveness (34). Therefore, B cell-mediated IgE responses serve as an important bridge connecting the type 2 cytokine network and allergic effector responses.

Eosinophils, mast cells, and basophils are important effector cells of type 2 inflammatory responses. IL-5 secreted by Th2 cells, ILC2s, and Tc2 cells promotes the recruitment, activation, and survival of eosinophils, while IL-13 induces airway epithelial cells to produce chemokines such as eotaxin, further enhancing eosinophil infiltration (18, 35). Activated eosinophils can release cytotoxic granule proteins, reactive oxygen species, and various inflammatory mediators, leading to epithelial damage and airway remodeling (35). Meanwhile, mast cells and basophils, as IgE-dependent effector cells, rapidly release inflammatory mediators upon re-exposure to allergens, participating in both acute asthma exacerbations and persistent inflammatory processes (34).

In addition, recent studies have found that other innate lymphoid cell subsets besides ILC2 may also be involved in the pathogenesis of asthma. For example, ILC3s can participate in airway inflammation and tissue remodeling by secreting IL-17 and IL-22, and have received increasing attention in neutrophilic asthma and glucocorticoid-resistant inflammation (36). However, their specific roles in classical type 2 asthma still require further elucidation.

In summary, iNKT cells, MAIT cells, γδT cells, dendritic cells, B cells, and a variety of effector cells collectively participate in the construction and regulation of the type 2 inflammatory network. Although all of the above-mentioned cells are involved in the regulation of type 2 inflammatory responses, current evidence indicates that Th2 cells, ILC2s, and Tc2 cells still constitute the core immune network driving the initiation, maintenance, and exacerbation of type 2 inflammation, and also represent the primary focus of current mechanistic research and targeted therapies. Therefore, this review focuses on the above three cell types and their related molecular mechanisms.

### 2.6 Involvement of airway epithelial cells and ferroptosis

Beyond immune cells, structural cells such as airway epithelial cells also actively participate in shaping the inflammatory microenvironment. Beyond immune cells, airway epithelial cells themselves play a crucial role in Th2 inflammation. Rather than serving merely as a passive physical barrier, airway epithelial cells act as active participants and initiators of immune responses. Upon stimulation and damage, these cells release alarmins such as interleukin-33 (IL-33) and thymic stromal lymphopoietin (TSLP), thereby initiating downstream immune responses (37). At the same time, epithelial cells themselves serve as key target cells in Th2 inflammation, as interleukin-13 (IL-13) can directly act on them to induce MUC5AC expression and drive mucus hypersecretion (38). The integrity of the epithelial barrier is essential for maintaining airway homeostasis; its disruption not only facilitates the entry of allergens and pathogens but also establishes a vicious cycle that amplifies inflammation.

Airway epithelial cells represent a key site for the occurrence of ferroptosis in asthma, and epithelial ferroptosis in clinical samples is closely associated with disease severity. Miao et al. reported that serum levels of the ferroptosis marker malondialdehyde (MDA) are significantly elevated in patients with T2 asthma compared with healthy controls, and are positively correlated with interleukin-13 (IL-13) levels and lung function parameters, such as forced expiratory volume in one second (FEV1%), suggesting that ferroptosis contributes to the pathophysiology of T2 asthma (39). Li et al. likewise reported that levels of carcinoembryonic antigen-related cell adhesion molecule 5 (CEACAM5) are elevated in induced sputum and serum from patients with asthma and are associated with markers of ferroptosis (40).

In animal models, the hallmarks of ferroptosis have been directly confirmed in airway epithelial cells. In an ovalbumin (OVA)-induced asthma mouse model, lung tissue exhibits increased MDA levels, a decreased reduced glutathione/oxidized glutathione (GSH/GSSG) ratio, and elevated iron levels, while electron microscopy reveals characteristic ferroptotic morphological changes in airway epithelial cell mitochondria, including shrinkage and reduced cristae. Notably, IL-13 deficiency reverses these alterations, indicating that IL-13 acts as a key upstream inducer of epithelial ferroptosis (39). Song et al. likewise observed the occurrence of epithelial ferroptosis in a house dust mite (HDM)-induced asthma model and confirmed that it is closely associated with the exacerbation of airway inflammation (41).

In addition, environmental factors can exacerbate asthma by inducing ferroptosis in airway epithelial cells. Chen et al. demonstrated that exposure to coke oven emissions (COE) synergizes with the allergen house dust mite (HDM) to induce ferroptosis in airway epithelial cells, as evidenced by increased lipid peroxidation and decreased expression of glutathione peroxidase 4 (GPX4) (42). Zhang et al. further demonstrated that exposure to fine particulate matter (PM2.5) can induce ferroptosis in airway epithelial cells by altering chromatin accessibility and upregulating the expression of ferritin heavy chain 1 (FTH1) and ferritin light chain (FTL) (43).

Collectively, these studies establish airway epithelial cells as a key site for ferroptosis in asthma. The occurrence of ferroptosis not only compromises epithelial barrier integrity but may also amplify inflammatory responses through the release of damage-associated molecular patterns (DAMPs), thereby creating a self-perpetuating vicious cycle. These findings link regulated cell death to Th2 inflammation and provide potential new therapeutic targets for asthma.

## 3 Key signaling pathways and molecular regulatory mechanisms

The activation and effector functions of the immune cells described above rely on precise intracellular signaling regulation. The JAK/STAT pathway, epigenetic modifications, ferroptosis-related pathways, and the p38 MAPK signaling pathway collectively constitute a regulatory network underlying Th2 inflammation. These pathways are relatively independent yet highly interconnected, jointly determining the magnitude and persistence of Th2 inflammatory responses.

Among these signaling cascades, the JAK/STAT pathway is most closely associated with Th2 inflammation. Kim et al. revealed a critical role for JAK3 in ILC2 activation and steroid resistance. They demonstrated that, under combined stimulation with IL-2, IL-7, and IL-33, STAT3, STAT5, and STAT6 are phosphorylated in ILC2s, with STAT5 activation being particularly essential. Notably, JAK3 inhibitors block the phosphorylation of these STAT proteins, induce apoptosis of ILC2s, and suppress their proliferation and cytokine production, whereas JAK1/2 inhibitors do not exert these effects, highlighting the nonredundant role of JAK3 in ILC2 signaling (20). This finding provides important insight into the mechanisms underlying steroid resistance in ILC2s.

Further studies have demonstrated that dysregulation of the JAK/STAT pathway is closely associated with steroid resistance. In an OVA/IL-33–induced steroid-resistant asthma mouse model, ILC2s acquire glucocorticoid resistance whereas Th2 cells remain steroid-sensitive, suggesting fundamental differences in their underlying signaling pathways. The JAK3/STAT5 axis plays a pivotal role in both ILC2 activation and the development of steroid resistance, and the emergence of multipotent ILC2s (IL-5^+^IL-13^+^IL-17A^+^) is closely linked to this phenotype; notably, JAK3 inhibitors can suppress the generation of these cells (20). IL-4 induces the expression of GATA3 via activation of STAT6, thereby promoting Th2 cell differentiation and the production of cytokines such as IL-5 and IL-13, and contributing to the pathogenesis of asthma. Mechanistically, IL-4 signals through the type I IL-4 receptor composed of IL-4Rα and the common γ chain (γc, also known as IL-2Rγ), leading to activation of the associated JAK1/JAK3–STAT6 signaling cascade. In contrast, IL-13 primarily signals through the type II IL-4 receptor consisting of IL-13Rα1 and IL-4Rα, which activates downstream JAK1/TYK2–STAT6 signaling (44).

SLC7A11 encodes the key functional subunit of system Xc^-^ (xCT) and is an important molecule for maintaining cellular antioxidant capacity and inhibiting ferroptosis. System Xc^-^ provides raw materials for glutathione (GSH) synthesis by mediating the uptake of extracellular cystine; GSH, in turn, is an essential cofactor for glutathione peroxidase 4 (GPX4) to exert its antioxidant function. GPX4 is able to scavenge intracellular lipid peroxides, thereby maintaining redox homeostasis and preventing ferroptosis.

**Figure 2:**
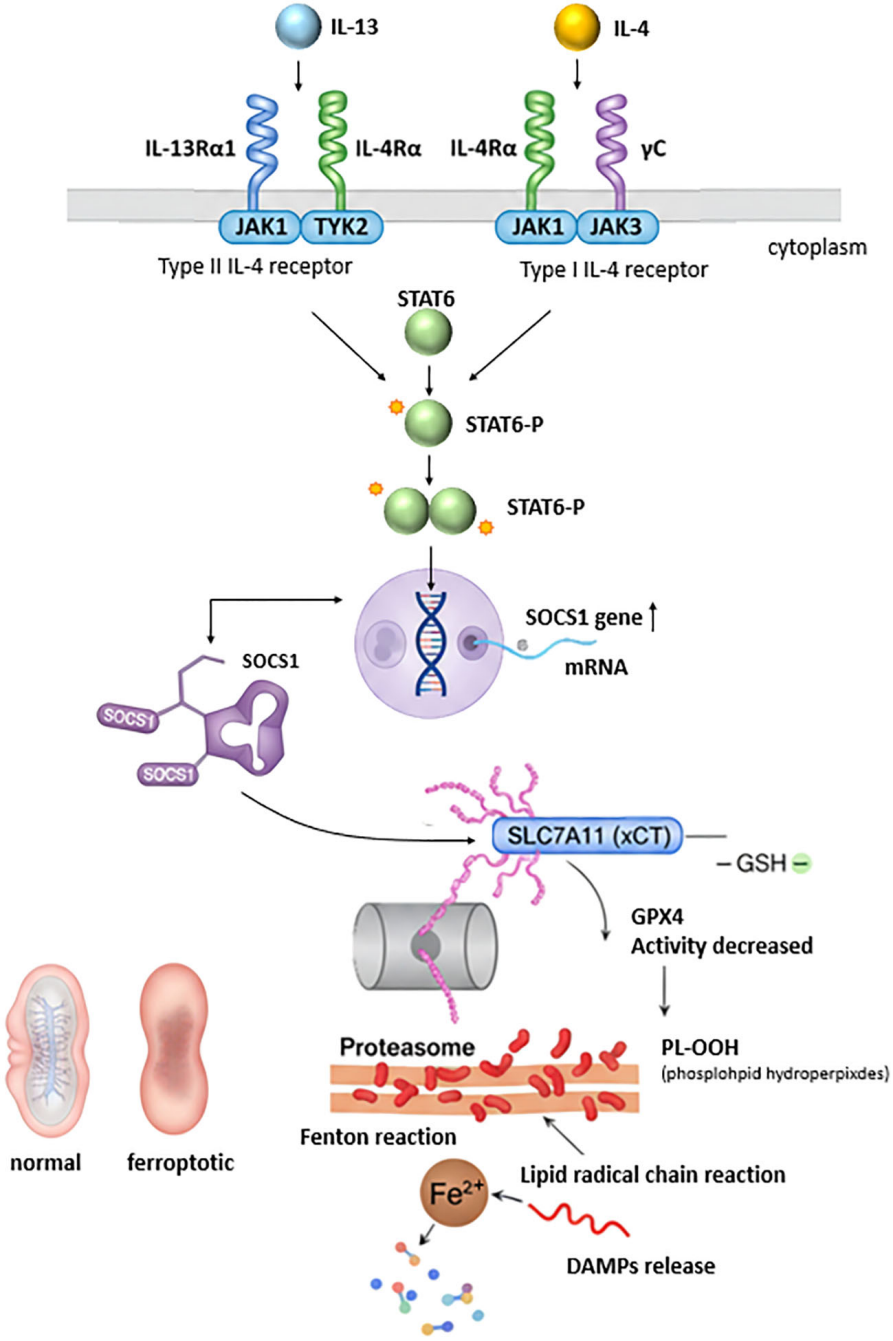
Schematic illustration of IL-4/IL-13 receptor signaling and the STAT6/SOCS1/SLC7A11-mediated ferroptotic pathway in airway epithelial cells. IL-13 signals through the type II IL-4 receptor complex composed of IL-13Rα1 and IL-4Rα, whereas IL-4 signals through the type I IL-4 receptor consisting of IL-4Rα and the common γ chain (γc, also known as IL-2Rγ). Activation of these receptor complexes leads to downstream JAK–STAT6 signaling, including JAK1/TYK2 activation in response to IL-13 and JAK1/JAK3 activation in response to IL-4. Phosphorylated STAT6 undergoes dimerization and translocates into the nucleus, where it promotes SOCS1 transcription. Increased SOCS1 expression facilitates the ubiquitination and proteasomal degradation of SLC7A11, the functional subunit of system Xc−, thereby impairing cystine uptake and reducing intracellular glutathione (GSH) levels. Consequently, GPX4 activity is diminished, resulting in the accumulation of phospholipid hydroperoxides (PL-OOH). In the presence of iron-dependent Fenton reactions, excessive lipid peroxidation promotes ferroptotic cell death, which is characterized by mitochondrial shrinkage and the release of damage-associated molecular patterns (DAMPs), thereby contributing to airway inflammation.

In the ferroptosis pathway, IL-13 can upregulate SOCS1 expression through STAT6 activation. SOCS1 further promotes the ubiquitination and degradation of SLC7A11, leading to impaired system Xc^-^ function, reduced cystine uptake, and decreased GSH synthesis, thereby weakening the ability of GPX4 to scavenge lipid peroxides. As lipid peroxides continue to accumulate, ferroptosis is eventually induced in cells. Since STAT6 activation depends on upstream JAK kinases (such as JAK1/JAK2/TYK2), this finding suggests that the JAK/STAT pathway may be involved in ferroptosis by regulating the SLC7A11-xCT-GSH-GPX4 antioxidant axis, further expanding the role of JAK/STAT signaling in the pathogenesis of asthma (39) (**Figure 2**). In addition to IL-13, IL-17A can also affect ferroptosis-related processes. Studies have found that IL-17A can disrupt the antioxidant defense system composed of xCT, GSH, and GPX4, reducing the ability of cells to scavenge lipid peroxides, thereby inducing ferroptosis in airway epithelial cells (41); furthermore, CEACAM5 can promote reactive oxygen species generation and lipid peroxidation by activating the JAK/STAT6 signaling pathway, further exacerbating ferroptosis and airway inflammation (45). Crosstalk exists between ferroptosis and autophagy: Zhao et al. reported that phosphatidylethanolamine-binding protein 1 (PEBP1) acts as a “rheostat” in determining whether cells undergo autophagy or ferroptosis (46), while Zeng et al. demonstrated that house dust mite (HDM)–induced ferroptosis is dependent on NCOA4-mediated ferritinophagy (47). Pan et al. further identified GABARAPL1 as an endogenous inhibitor of ferroptosis (48).

Epigenetic modifications also play a critical role in the regulation of Th2 inflammation. In particular, DNA methylation can stably modulate gene expression without altering the underlying DNA sequence, thereby mediating the effects of environmental exposures on asthma risk (49, 50). Early-life environmental exposures, such as farm environments, air pollution, and prenatal famine, can induce persistent changes in DNA methylation, which may accumulate across the lifespan and influence disease susceptibility (51). For example, the protective effect of farm exposure has been associated with DNA methylation changes in the TNFAIP3 gene, an effect that is more pronounced in children carrying specific genetic variants (52). These findings are consistent with results from large-scale genetic studies. Moffatt et al., through a genome-wide association study (GWAS), identified multiple loci significantly associated with asthma, including IL33, IL1RL1/IL18R1, SMAD3, IL2RB, and the HLA-DQ region, highlighting the critical roles of epithelial injury signaling to the adaptive immune system and the activation of airway inflammation in asthma pathogenesis. Notably, the study also found limited overlap between genetic loci associated with total serum IgE levels and those linked to asthma susceptibility, suggesting that elevated IgE may represent a secondary phenomenon rather than a primary driver of the disease (15). This conclusion is consistent with the concept that environmental factors influence asthma susceptibility through epigenetic mechanisms, further indicating that the development of asthma results from the combined effects of genetic predisposition, environmental exposures, and epigenetic regulation.

The p38 MAPK pathway also plays an important role in the regulation of Th2 inflammation. Endo et al. demonstrated that interleukin-33 (IL-33) confers pathogenicity to memory Th2 cells via the p38 MAPK signaling pathway. Inhibition of p38 MAPK suppresses the production of IL-5 and IL-13 by pathogenic Th2 (pTh2) cells without affecting GATA3 expression. Notably, this pathway operates independently of the canonical STAT6 pathway, providing a novel strategy for targeting pTh2 cells (11). Khan et al. further confirmed that, in a model of pTh2 differentiation induced by thymic stromal lymphopoietin (TSLP) in combination with a GITR agonist, p38 MAPK inhibition significantly suppresses the production of IL-5 and IL-13, whereas AP-1 inhibition does not exert this effect (6). This finding suggests that the p38 MAPK pathway may represent a more specific regulatory target for pathogenic Th2 (pTh2) cells.

It is noteworthy that HIF-2α–mediated metabolic reprogramming has become a recent research hotspot. Zou et al. found that HIF-2α is selectively highly expressed in pathogenic Th2 cells, where it promotes phosphatidylinositol-3,4,5-trisphosphate(PIP3) synthesis by regulating inositol polyphosphate multikinase (IPMK) expression, thereby enhancing the activity of the TCR–PI3K–AKT signaling pathway. The HIF-2α–specific inhibitor PT-2385 significantly alleviates airway inflammation in asthmatic mouse models and reduces the proportion of pathogenic Th2 cells (7). This finding directly links metabolic regulation to the pathogenicity of Th2 cells, revealing a novel therapeutic target. Interestingly, metabolic reprogramming is closely interconnected with the signaling pathways mentioned above— the PI3K–AKT pathway can influence mTOR activity to further regulate cellular metabolism, while metabolic status can in turn feedback to modulate the activity of pathways such as JAK/STAT, thereby forming a complex regulatory network.

In addition to epithelial cell–centered signaling cascades such as IL-13–induced ferroptosis, steroid-resistant mechanisms in innate and adaptive type 2 lymphocytes are increasingly recognized as key drivers of severe asthma.

**Figure 3:**
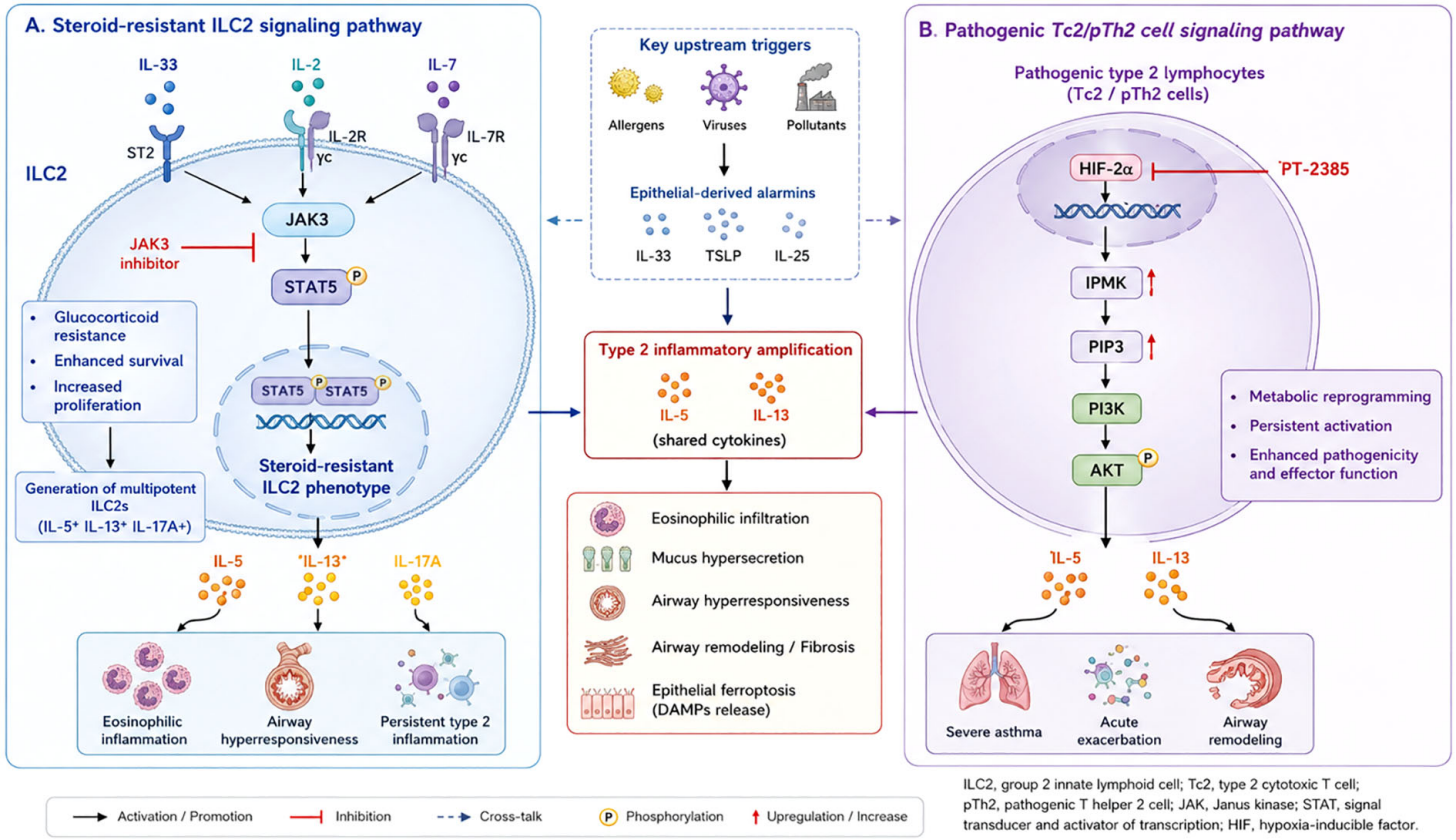
Pathogenic signaling pathways in steroid-resistant ILC2s and pathogenic type 2 lymphocytes in severe asthma. This schematic illustrates two key intracellular signaling axes involved in severe type 2 asthma. **(A)** Steroid-resistant ILC2 signaling pathway: epithelial-derived alarmins (IL-33, TSLP, and IL-25) activate ILC2s through cytokine receptors, inducing JAK3 activation and downstream STAT5 phosphorylation. Persistent STAT5 signaling promotes steroid-resistant ILC2 phenotypes characterized by enhanced survival, proliferation, and increased production of IL-5, IL-13, and IL-17A. Pharmacological inhibition of JAK3 suppresses this pathway and restores glucocorticoid responsiveness. **(B)** Pathogenic Tc2/pTh2 cell signaling pathway: HIF-2α-mediated metabolic reprogramming promotes IPMK expression, PIP3 accumulation, and activation of the PI3K-AKT signaling pathway in pathogenic type 2 lymphocytes. This signaling axis enhances effector cytokine production (IL-5 and IL-13), contributing to severe asthma, acute exacerbations, and airway remodeling. Pharmacological inhibition of HIF-2α (e.g., PT-2385) attenuates this pathogenic cascade. Together, these pathways highlight distinct but complementary mechanisms driving steroid resistance and severe type 2 inflammation.

As illustrated in **Figure 3**, steroid-resistant ILC2 activation is mediated by the JAK3/STAT5 signaling axis downstream of IL-2, IL-7, and IL-33 stimulation. Persistent STAT5 activation promotes enhanced survival, proliferation, and cytokine production (IL-5, IL-13, and IL-17A), contributing to glucocorticoid resistance.

In parallel, pathogenic type 2 lymphocytes (Tc2 and pathogenic Th2 cells) exhibit metabolic reprogramming driven by HIF-2α signaling. HIF-2α promotes IPMK expression and activates the PI3K–AKT pathway, sustaining effector cytokine production and driving severe asthma progression.

## 4 Therapeutic targets and intervention strategies for asthma

An in-depth understanding of the aforementioned immune cell subsets, alarmin systems, and intracellular signaling networks is reshaping the clinical intervention landscape for severe asthma. Treatment strategies are shifting from traditional non-specific immunosuppression toward precise interventions targeting specific molecular nodes and cell populations.

### 4.1 Clinical biologics targeting type 2 inflammation

In recent years, highly specific monoclonal antibodies (mAbs) developed against different axes of the Type 2 (T2) inflammatory cascade have emerged as the cornerstone of precision therapy for severe, uncontrolled asthma. Based on the biological targets they intercept, currently clinically approved biologics are principally categorized into the following four major classes.

Upstream alarmin blockade: Thymic stromal lymphopoietin (TSLP) is one of the earliest alarmins released by the airway epithelium upon stimulation by allergens, viruses, or pollutants, positioning it at the absolute upstream of the Type 2 inflammatory cascade. Tezepelumab, the first human monoclonal antibody targeting TSLP, intercepts TSLP signaling at the very inception of inflammatory initiation, thereby broadly suppressing downstream Type 2 immune responses mediated by ILC2s, Th2, and Tc2 cells (53). Distinct from biologics that merely target a single effector cytokine, tezepelumab simultaneously encompasses both T2-high and certain non-T2 inflammatory pathways (53). The Phase III NAVIGATOR clinical trial demonstrated that in patients with uncontrolled severe asthma, tezepelumab reduced the annualized asthma exacerbation rate (AAER) by 56%, independent of baseline blood eosinophil counts or fractional exhaled nitric oxide (FeNO) levels (28), fully underscoring its broad-spectrum anti-inflammatory advantages.

Core cytokine receptor antagonism: IL-4 and IL-13 are the central effector cytokines driving Type 2 inflammation, both sharing the IL-4 receptor alpha subunit (IL-4Rα) for signal transduction (53). Dupilumab, a fully human monoclonal antibody targeting IL-4Rα, simultaneously blocks both IL-4 and IL-13 signaling (54). This dual blockade mechanism not only inhibits IgE class switching in B cells but, more crucially, acts directly on airway epithelial cells to reverse the previously described IL-13-induced ubiquitination and degradation pathway of SOCS1/SLC7A11. By restoring SLC7A11 expression and glutathione synthesis, it suppresses epithelial cell ferroptosis, thereby preserving the integrity of the airway barrier (54). In the Phase III QUEST trial, dupilumab reduced the AAER by approximately 48% in patients with moderate-to-severe asthma and significantly improved lung function (54). For patients with oral corticosteroid (OCS)-dependent severe asthma, dupilumab also exhibited a remarkable OCS-sparing effect (55). Its efficacy is not strictly restricted by baseline blood eosinophil counts, though patients with FeNO ≥25 ppb or blood eosinophils ≥150/μL derive the most pronounced benefit (55).

Anti-eosinophil pathway strategies: Eosinophilic infiltration is the central pathological hallmark of T2-high asthma airway inflammation, and IL-5 serves as the critical cytokine regulating eosinophil differentiation, activation, and survival (53). Mepolizumab and reslizumab directly bind to and neutralize free IL-5, blocking eosinophil production and activation, thereby reducing airway eosinophilic infiltration (53). In patients with severe eosinophilic asthma, mepolizumab reduced the AAER by approximately 49% (IRR 0.49, 95% CI 0.38-0.66) (53). Benralizumab, on the other hand, targets the IL-5 receptor alpha subunit (IL-5Rα) expressed on the surface of eosinophils, and directly clears tissue eosinophils via natural killer (NK) cell-mediated antibody-dependent cellular cytotoxicity (ADCC), achieving near-complete eosinophil depletion (56). In the two landmark Phase III trials, SIROCCO and CALIMA, benralizumab significantly reduced the AAER in severe asthma patients with blood eosinophils ≥300/μL (51% in SIROCCO and 28% in CALIMA, respectively) (56, 57). The MIRACLE trial in an Asian population further confirmed that benralizumab reduced the AAER by 74% (RR 0.26, 95% CI 0.19-0.36) (58). Notably, benralizumab can also rapidly restore the glucocorticoid sensitivity of ILC2s, a finding that closely echoes the molecular mechanisms of ILC2 steroid resistance described in Chapter 3 of this review.

Classical IgE cascade interception: IgE acts as a pivotal bridging molecule for allergen-triggered acute airway inflammatory responses. Omalizumab, a humanized monoclonal antibody targeting free IgE, effectively prevents IgE from binding to the high-affinity receptor FcϵRI on the surface of mast cells and basophils, thereby blocking allergen-induced release of acute inflammatory mediators at its source (53). In patients with allergic asthma, omalizumab significantly reduced the AAER (IRR 0.56, 95% CI 0.40-0.77) (53). This therapy is indicated for patients with allergic asthma whose serum total IgE levels range between 30 and 700 IU/mL, and its clinical efficacy correlates positively with baseline blood eosinophil counts (59).

The clinical selection among these four categories of biologics necessitates a comprehensive consideration of patient-specific biomarker profiles (blood eosinophil counts, FeNO levels, and IgE levels), asthma phenotypes, comorbidities, and historical treatment responses, ultimately fulfilling the precision medicine objective of delivering “the most appropriate biologic for the right patient.” (**Table 2**).

**Table 2:** Overview of clinically approved monoclonal antibodies, their molecular targets, and landmark clinical trials for severe type 2 asthma.

Biologic	Molecular target	MOA	Indicated asthma phenotype
Omalizumab	IgE	Blocks IgE binding to mast cells and basophils	Severe allergic asthma
Mepolizumab	IL-5	Neutralizes free IL-5 to inhibit eosinophil proliferation	Severe eosinophilic asthma
Reslizumab	IL-5	Neutralizes free IL-5 to reduce eosinophilic infiltration	Severe eosinophilic asthma
Benralizumab	IL-5Rα	Targets IL-5Rα to induce apoptosis via ADCC effect	Severe eosinophilic asthma
Dupilumab	IL-4Rα	Simultaneously blocks the shared receptor pathway of IL-4 and IL-13	T2-high/moderate-to-severe asthma with nasal polyps
Tezepelumab	TSLP	Targets the ultimate upstream alarmin to broadly suppress Type 2 immune responses	Broad-spectrum severe asthma (regardless of T2-high/low expression)

TSLP, thymic stromal lymphopoietin; IL, interleukin; IL-4Rα, interleukin-4 receptor alpha subunit; IgE, immunoglobulin E; FcϵRI, high-affinity IgE receptor; mAb, monoclonal antibody; FeNO, fraction of exhaled nitric oxide; EOS, blood eosinophil count; OCS, oral corticosteroids; FDA, U.S. Food and Drug Administration. a Landmark Phase III clinical trials demonstrating significant reduction in annualized asthma exacerbation rates (AAER) and/or OCS sparing effects. b Dupilumab is uniquely approved for both moderate-to-severe asthma and severe chronic rhinosinusitis with nasal polyps (CRSwNP), targeting the shared IL-4/IL-13 signaling pathway in the continuous airway.

### 4.2 Intracellular signaling pathway inhibitors and small-molecule interventions

Among these, JAK3 inhibitors have been the most extensively studied, with their mechanisms of action relatively well characterized. Kim et al. demonstrated that the JAK3 inhibitor PF-06651600 induces apoptosis in ILC2s, while also suppressing their proliferation and cytokine production. In a steroid-resistant asthma mouse model, JAK3 inhibition restored the glucocorticoid sensitivity of ILC2s and reduced the generation of multipotent ILC2s (IL-5^+^IL-13^+^IL-17A^+^). Mechanistic studies further revealed that JAK3 inhibitors exert their effects by blocking the phosphorylation of STAT3, STAT5, and STAT6, with minimal impact on JAK1/2 signaling. Notably, the combination of JAK3 inhibitors with glucocorticoids has shown strong therapeutic efficacy in steroid-resistant asthma. This synergistic effect is based on complementary targeting of key effector cell populations: glucocorticoids primarily suppress Th2 cells, whereas JAK3 inhibitors act on ILC2s, together covering the two major effector arms of type 2 inflammation. In an OVA/IL-33-induced steroid-resistant asthma model, combination therapy significantly reduced airway hyperresponsiveness and eosinophilic infiltration (20).

In addition to the prominent role of JAK3 inhibitors in ILC2 regulation, JAK1 inhibitors have also demonstrated broad prospects in the therapeutic landscape of allergic diseases. Atopic dermatitis (AD) and asthma are both common Type 2 inflammatory barrier diseases that share a highly similar core pathogenesis; both are characterized by epithelium-derived alarmins (IL-33, TSLP) driving downstream Th2/ILC2 immune responses, and their key pathogenic cytokines—including IL-4, IL-13, and IL-31—rely stringently on the JAK/STAT pathway for signal transduction. Consequently, the successful application of JAK inhibitors in AD provides a pivotal reference paradigm for parallel therapeutic strategies in asthma. Currently, the oral selective JAK1 inhibitors abrocitinib and upadacitinib have been approved by the U.S. Food and Drug Administration (FDA) for the treatment of moderate-to-severe AD, while topical ruxolitinib cream (a JAK1/2 inhibitor) has been approved for mild-to-moderate AD (60, 61). Characterized by a rapid onset of action, these agents swiftly alleviate pruritus and improve skin lesions, with their clinical efficacy robustly validated across multiple Phase III randomized controlled trials (62).

In the field of asthma, early proof-of-concept studies on inhaled JAK inhibitors have likewise yielded encouraging results. Phase I clinical trials of GDC-0214 and GDC-4379 confirmed that inhaled JAK1 inhibitors could significantly reduce fractional exhaled nitric oxide (FeNO) levels and peripheral blood eosinophil counts in patients with mild asthma (63, 64). However, while another inhaled JAK1 inhibitor, AZD0449, exhibited favorable lung retention and an acceptable safety profile in a Phase I study, it failed to demonstrate a significant reduction in FeNO compared with placebo within the 1.2–5.0 mg dose range; this discrepancy underscores that the free drug concentration within the target tissue represents a critical bottleneck limiting the development of inhaled JAK inhibitors (65). Building upon these insights, a next-generation inhaled selective JAK1 inhibitor, londamocitinib (AZD4604), is currently advancing through clinical development. The ongoing ARTEMISIA study (NCT06435273) is a Phase II mechanism-validation trial in patients with moderate-to-severe asthma. It focuses not only on canonical Type 2 inflammatory pathways but also specifically targets non-Type 2 and glucocorticoid-insensitive inflammatory mechanisms (such as the IL-6/STAT3, IFN-γ/STAT1, and IL-17-related pathways), aiming to elucidate the molecular signatures of pulmonary JAK1 inhibition via multi-omic analysis (66).

Targeted therapies against pathogenic Th2 (pTh2) cells are also under active investigation. Khan et al. found that TSLP and GITR/4-1BB costimulatory signals synergistically induce pTh2 cell differentiation—TSLP acts through the STAT5 pathway, while GITR/4-1BB enhances Th2 cytokine expression via the NF-κB and MAPK pathways. Combined blockade of these signaling axes can effectively suppress the generation of pTh2 cells (6). Bick et al. further demonstrated that IL-9 and IL-21 play complementary roles in asthma, and that their combined blockade can synergistically reduce airway inflammation (67). The selective effects of p38 MAPK inhibitors make them promising therapeutic agents. Specifically, p38 MAPK inhibitors can selectively suppress IL-5 and IL-13 production in pTh2 cells without affecting IL-4 expression or GATA3 levels. This selectivity suggests that they may inhibit the effector functions of pathogenic Th2 subsets without impairing the overall functionality of Th2 cells.

### 4.3 Emerging targets and atypical intervention strategies

The discovery of the ferroptosis pathway has provided a novel therapeutic target for asthma treatment. Targeting SOCS1 may inhibit epithelial cell ferroptosis. SOCS1 knockdown blocks IL-13–induced downregulation of SLC7A11 and suppresses ferroptosis, suggesting that SOCS1 inhibitors may help preserve airway epithelial barrier function and improve airway hyperresponsiveness. In an ovalbumin-induced asthma mouse model, SOCS1 inhibition reduced airway inflammation and epithelial injury. Meanwhile, enhancing SLC7A11 function also represents a potential therapeutic strategy, as overexpression of SLC7A11 can reverse the pro-ferroptotic effects of IL-13. The glutathione precursor N-acetylcysteine is already used clinically, and its ability to inhibit ferroptosis may partly account for its therapeutic efficacy (39). Bao et al. reported that the ferroptosis inhibitor liproxstatin-1 can alleviate bronchial epithelial cell injury and neutrophilic asthma induced by LPS/IL-13 (68).

HDAC1, as a negative regulator of Th2 pathogenicity, has attracted increasing attention in the development of activators. Khan et al. demonstrated that HDAC1 functions as a suppressor of pathogenic Th2 (pTh2) cell differentiation, and that enhancing HDAC1 activity may inhibit pTh2 pathogenicity and reduce the production of IL-5 and IL-13. In HDAC1-deficient mice, the proportion of pTh2 cells is increased and their pathogenicity is enhanced, suggesting that maintaining HDAC1 function is critical for controlling Th2-mediated inflammation (6). However, the application of natural HDAC inhibitors remains controversial. Short-chain fatty acids (SCFAs), as endogenous HDAC inhibitors, show contradictory effects in asthma: on one hand, they may enhance anti-inflammatory responses by inhibiting HDAC activity; on the other hand, they may also promote the expression of Th2 cytokines. These findings suggest that more selective strategies targeting specific HDAC isoforms are required (6). Studies have shown that different members of the HDAC family play opposing roles in immune regulation. Therefore, developing selective HDAC1 activators, rather than broad-spectrum HDAC inhibitors, may represent a more favorable therapeutic strategy.

Precision targeted therapy based on immune cell surface markers represents a promising future direction. Markers such as ST2^+^CD27^-^KLRG1^-^ can be used to identify and isolate pTh2 cells (11), while CD45RO can serve as a marker for inflammatory ILCs (9). Antibody-based or cell-based therapeutic strategies targeting these markers warrant further exploration. Allergen-specific immunotherapy (AIT), the only treatment currently capable of modifying the course of allergic diseases, has also gained new mechanistic insights in recent years. Shamji et al. summarized the immunological mechanisms of AIT, highlighting that it not only induces regulatory T (Treg) cells and IgG4 production but also promotes regulatory ILC2 (ILC2reg) while suppressing Th2A and Tfh cells (69). Golebski et al. reported that a population of IL-10–producing ILC2s emerges after AIT, with levels correlating with clinical improvement (70). Sharif et al. further demonstrated that AIT reduces circulating Tfh cells while increasing Tfr cells and IL-10^+^ Tfh cells, accompanied by changes in chromatin accessibility (41). In addition, Shamji et al. found that subcutaneous immunotherapy (SCIT) primarily induces IgG4 production, whereas sublingual immunotherapy (SLIT) preferentially promotes IgA1/2 responses. These antibodies contribute to allergen neutralization at the nasal mucosal surface (71).

## 5 Challenges and future directions

Despite substantial progress in the study of T2 asthma, numerous challenges remain. Addressing the limitations of current research, identifying key future directions, and exploring feasible pathways for clinical translation are essential for advancing the field.

First, the epigenetic mechanisms underlying steroid resistance in ILC2s have not yet been fully elucidated. Although the involvement of the JAK3/STAT5 pathway has been established, chromatin-level alterations, histone modifications, and the regulatory roles of non-coding RNAs require further in-depth investigation (20). Elucidation of these mechanisms will provide a theoretical basis for the development of more specific therapeutic strategies. Second, the mechanisms underlying functional crosstalk among immune cell subsets remain incompletely understood—how Th2 cells, ILC2s, and Tc2 cells coordinate *in vivo*, and how intercellular communication networks are organized, require further investigation (13). The application of single-cell and spatial omics technologies is expected to elucidate these complex interactions. Third, although the role of ferroptosis in asthma has been primarily demonstrated in animal models, its relevance in human asthma requires further clinical evidence, including large-scale cohort studies and interventional clinical trials (39). Fourth, although the heterogeneity of pathogenic Th2 (pTh2) cells has been recognized, whether distinct subsets exhibit interconversion and how they respond to therapeutic interventions remain to be elucidated (6).

In future research, the dynamic regulation of the asthma immune network will remain a major focus. Single-cell and spatial transcriptomics technologies provide powerful tools for dissecting immune cell interactions. He et al. employed spatial transcriptomics to characterize the spatial microenvironment of T cell differentiation within asthmatic lymph nodes and found that interleukin-2 (IL-2) forms a localized niche that drives the early differentiation of Blimp-1^+^ Th2 cells (72). Similar studies will help elucidate the dynamic regulatory mechanisms of immune cells *in vivo*; when integrated with time-series analyses and lineage tracing approaches, they are expected to enable the construction of a comprehensive spatiotemporal map of the asthma immune response.

The development of novel targeted therapies requires a careful balance between specificity and safety. Compared with broad-spectrum JAK inhibitors, JAK3 inhibitors offer greater selectivity; however, their pharmacokinetic properties and long-term safety profiles still require further optimization (20). HIF-2α inhibitors target pathogenic Th2 cells and may therefore offer a favorable safety profile; however, their clinical efficacy in humans remains to be validated (7). SOCS1 inhibitors, as a novel therapeutic target, require further preclinical studies to support their development (39).

Beyond small-molecule inhibitors and biologic therapies, engineered immune-cell approaches are emerging as a promising therapeutic modality for severe and refractory T2 asthma. Recently, Jin et al. developed long-lived multifunctional CAR-T cells capable of simultaneously targeting eosinophilic inflammation and suppressing IL-4/IL-13 signaling, resulting in sustained disease remission in multiple experimental asthma models (73). In addition, advances in CAR-engineered regulatory T cells (CAR-Tregs) have highlighted their potential to restore immune tolerance and suppress pathogenic immune responses in allergic and inflammatory diseases (74). Although challenges related to safety, target selection, manufacturing complexity, and clinical translation remain, CAR-based cellular immunotherapy may represent a next-generation precision medicine strategy capable of achieving durable disease modification beyond the limitations of current biologics.

The establishment of personalized therapeutic strategies relies on robust and reliable biomarkers. The proportion of CD45RO^+^ ILC2s can predict the risk of steroid resistance (19), serum MDA levels reflect the extent of ferroptosis (39), and hypoxia-inducible factor 2α (HIF-2α) expression indicates the activity of pathogenic Th2 responses (7). These biomarkers may help guide treatment selection. In addition, genetic polymorphisms (e.g., at IL1RL1 and TSLP loci) may predict patient responses to specific biologic therapies (15). The integration of multi-omics data to establish a molecular classification system for asthma will further advance the implementation of precision medicine. Moreover, deeper functional characterization of immune cell subsets is required; for newly identified populations—such as inflammatory ILC2s (19), multipotent ILC2s (20), and pTh2 subsets (6) —their functional properties and regulatory mechanisms need to be elucidated in greater detail, including their dynamic changes across disease stages, interactions with other cell types, and responses to therapeutic interventions.

The translation from basic research to clinical application faces multiple challenges. First, the differences between animal models and human disease must be carefully considered—although murine models can recapitulate certain features of human asthma, they differ substantially in immune system composition, airway structure, and disease progression. Second, the validation of biomarkers requires large-scale, multicenter clinical studies to establish standardized detection methods and reference ranges. Third, the safety and efficacy of novel targeted therapies must be rigorously evaluated through clinical trials, which typically demand substantial time and resources. Despite these challenges, emerging targets such as JAK3 and hypoxia-inducible factor 2α (HIF-2α) inhibitors have demonstrated translational potential and warrant further investigation. Notably, the successful approval of tezepelumab, a monoclonal antibody targeting thymic stromal lymphopoietin (TSLP), provides proof-of-concept for therapeutic strategies directed against upstream alarmins (28). Similarly, antibodies targeting the IL-33/ST2 axis have demonstrated promising efficacy in multiple clinical trials (37). These advances indicate that mechanism-based targeted therapies are progressively translating from bench to bedside.

## 6 Conclusion

In conclusion, Type 2 (T2) asthma is far more than a localized lesion driven by the activation of a single immune cell type; rather, it represents a dynamic, networked disease initiated by epithelial alarmins, orchestrated by multicellular synergy, and intertwined with multidimensional intracellular signaling pathways. Over the past decades, scholarly exploration has propelled two fundamental paradigm shifts in the medical understanding of this field. First, a shift from “single-cell-driven pathogenesis” toward “spatiotemporal immune network orchestration.” Although canonical pathogenic Th2 cells remain the cornerstone for maintaining chronic adaptive immunity and immunological memory, it is now well established that the rapid responsiveness and steroid-resistant nature of innate ILC2s, coupled with the non-canonical roles of Tc2 cells during severe and acute exacerbations, collectively constitute an indivisible pro-inflammatory axis.

Second, and perhaps of the greatest frontier academic value, is the paradigm shift from “conventional immune inflammation” toward a “vicious cycle of regulated cell death and barrier disruption.” The discovery of atypical mechanisms, such as ferroptosis, has shattered the historical perception of epithelial cells as mere “target organs of inflammation” or “passive physical barriers.” Compelling evidence that IL-13 mediates epithelial ferroptosis via intracellular ubiquitination pathways suggests that regulated cell death is not only a pathological consequence of Type 2 inflammation, but the damage-associated molecular patterns (DAMPs) it releases also serve as critical upstream triggers that amplify and sustain downstream immune networks. This vicious feedback loop of “immune activation—ferroptosis—barrier destruction—inflammatory amplification” highly likely represents the fundamental biological mechanism underlying refractory, steroid-resistant severe asthma.

Viewed through this lens of network resonance and cross-regulation, the future landscape of asthma intervention is poised for a comprehensive reshaping. Conventional, non-specific immunosuppression can no longer meet clinical demands. The future of precision medicine will transition from intercepting a single downstream effector (e.g., IL-5 or IgE) to focusing on two strategic domains: (1) targeting upstream initiation hubs (e.g., TSLP, IL-33) or key pluripotency nodes (e.g., JAK3, HIF-2α, SOCS1) to achieve a “package” blockade of multiple inflammatory pathways; and (2) protecting the epithelial barrier (e.g., via ferroptosis inhibition or HDAC1 activation) to sever the vicious cycle of inflammatory amplification at its source. Ultimately, the deep integration of single-cell spatial transcriptomics and multi-omic clinical molecular phenotyping will enable the dynamic decoding of patient-specific “immune-death” network landscapes. This will decisively propel asthma management from the era of “different treatments for the same disease” into an era of truly precise, individualized cures.
